# Diacetyloxyl derivatization of the fibroblast growth factor inhibitor dobesilate enhances its anti-inflammatory, anti-angiogenic and anti-tumoral activities

**DOI:** 10.1186/s12967-015-0413-4

**Published:** 2015-02-01

**Authors:** Javier Angulo, Pedro Cuevas, Begoña Cuevas, Mohammad El Youssef, Argentina Fernández, Eduardo Martínez-Salamanca, Rocío González-Corrochano, Guillermo Giménez-Gallego

**Affiliations:** Instituto Ramón y Cajal de Investigación Sanitaria (IRYCIS), Hospital Universitario Ramón y Cajal, Madrid, Spain; Departamento de Estructura y Función de Proteínas, Centro de Investigaciones Biológicas, Consejo Superior de Investigaciones Científicas, Avda Ramiro de Maeztu 9, ES-28040 Madrid, Spain

**Keywords:** Angiogenesis, Cancer, Cyclooxygenase, Dobesilate, Fibroblast growth factor, Inflammation

## Abstract

**Background:**

Dobesilate (2,5-dihydroxyphenyl sulfonate, DHPS) was recently identified as the most potent member of a family of fibroblast growth factor (FGF) inhibitors headed by gentisic acid, one of the main catabolites of aspirin. Although FGFs were first described as inducers of angiogenesis, they were soon recognized as broad spectrum mitogens. Furthermore, in the last decade these proteins have been shown to participate directly in the onset of inflammation, and their potential angiogenic activity often contributes to the inflammatory process *in vivo*. The aim of this work was to evaluate the anti-inflammatory, anti-angiogenic and anti-tumoral activities of the derivative of DHPS obtained by acetoxylation of its two hydroxyl groups (2,5-diacetoxyphenyl sulfonate; DAPS).

**Methods:**

Anti-inflammatory, anti-angiogenic and anti-tumoral activities of DHPS and DAPS were compared using *in vivo* assays of dermatitis, angiogenesis and tumorigenesis. The effects of both compounds on myeloperoxidase (MPO) and cyclooxygenase (COX) activities, cytokine production and FGF-induced fibroblast proliferation were also determined.

**Results:**

Topical DAPS is more effective than DHPS in preventing inflammatory signs (increased vascular permeability, edema, leukocyte infiltration, MPO activation) caused by contact dermatitis induction in rat ears. DAPS, but not DHPS, effectively inhibits COX-1 and COX-2 activities. DAPS also reduces the increase in serum cytokine concentration induced by lipopolysaccharide in rats. Furthermore, DAPS displays higher *in vivo* efficacy than DHPS in inhibiting FGF-induced angiogenesis and heterotopic glioma progression, with demonstrated oral efficacy to combat both processes.

**Conclusions:**

By inhibiting both FGF-signaling and COX-mediated prostaglandin synthesis, DAPS efficiently breaks the vicious circle created by the reciprocal induction of FGF and prostaglandins, which probably sustains undesirable inflammation in many circumstances. Our findings define the enhancement of anti-inflammatory, anti-angiogenic and anti-tumoral activities by diacetyloxyl derivatization of the FGF inhibitor, dobesilate.

## Introduction

Prostaglandins (PGs) are local mediators of inflammation, the synthesis of which is controlled by cyclooxygenases (COXs). The COX-1 isoform is constitutively expressed while COX-2 expression is induced in specific pathophysiological conditions or in response to inflammatory stimuli [1]. Non-steroidal anti-inflammatory drugs (NSAIDs), such as acetylsalicylic acid (ASA; aspirin), achieve their anti-inflammatory effects, at least in part, by inhibiting prostaglandin production [2]. Indeed, ASA irreversibly inhibits COX activity by acetylating a serine residue at the active centre of the enzyme [3]. Upregulation of COX-2 occurs in many diseases in which chronic inflammation plays a key role. Moreover, COXs have been widely implicated in tumorigenesis, a process to which they contribute by upregulating the expression of pro-angiogenic cytokines, vascular endothelial cell growth factor (VEGF) and fibroblast growth factor (FGF) [4-7], making them responsible for the pathological neovascularization of tumors. VEGF and FGF can also induce the expression of COX-2 and phospholipase A2, as well as the ensuing production of PG in endothelial cells, creating a positive feedback loop typical of chronic diseases [8,9].

Dobesilate (2,5-dihydroxyphenyl sulfonate, DHPS) is a small synthetic molecule with a good safety profile that has been widely used to treat diabetic retinopathy and chronic venous insufficiency [10,11], although its mechanism of action remained unknown and its efficacy has been criticized [12]. DHPS was rediscovered as the most active member of a family of compounds headed by gentisic acid, the catabolite of aspirin, which inhibit FGF-induced cell proliferation, migration and angiogenesis in distinct biological scenarios. These inhibitors were recently shown to interact with the heparin-binding domain of representative members of the FGF family and with FGF receptors (FGFRs), inducing structural alterations that block their interaction with heparin, and that interfere with the formation of the complex that triggers physiological cell responses to FGF [13].

Although initially classified as a broad-spectrum mitogen, FGF is now also believed to trigger inflammation, and it is closely associated with the inflammasome, which promotes neovascularization by rapidly inducing an inflammatory phenotype in microvascular endothelial cells [14,15]. Either directly or through FGF inhibition [16] DHPS also inhibits VEGF activity [17] and, accordingly, DHPS should also interfere with the PG/FGF-VEGF feedback loop described above. Indeed, these antagonistic effects should be taken into account when considering the preclinical efficacy of DHPS in treating gliomas [13,18], and the clinical benefits of using DHPS in humans to treat ocular inflammatory diseases [19,20], allergic contact dermatitis [21] and pathologies related to angioproliferative conditions, such as psoriasis [22] and basal cell carcinoma [23].

Gliomas are devastating brain tumors with exacerbated proliferation, angiogenesis and invasiveness [24] that display poor prognosis even after the introduction for its treatment of temozolomide in addition to radiotherapy [25]. Thus, the development of new strategies seems definitely appropriate. Considering the key role of inflammation in angiogenesis [26] and glioma [27,28], we explored whether anti-inflammatory properties could be added to drugs that interfere with glioma growth by inhibiting FGF [18] which may allow a better management of gliomas, as well as of other angiogenesis-dependent diseases. The search for novel anti-inflammatory drugs has identified many acetyloxyphenyl compounds that are effective inhibitors of COX activity [29,30]. DHPS contains a phenyl ring with two hydroxyl groups, which is readily accessible to introduce acetyloxyl groups, which should confer chemical properties similar to those of the aforementioned acetyloxyphenyl chemicals. With these premises our aim was to evaluate the anti-inflammatory activities of the acetyloxyl derivative of DHPS, 2,5-diacetyloxyphenyl sulfonate (DAPS) in *in vivo* and *in vitro* assays and its ability to inhibit angiogenesis and heterotopic glioma progression in animal models. Accordingly, we show that the acetoxylation of DHPS yields a compound with enhanced anti-inflammatory activity in different *in vivo* and *in vitro* circumstances, as well as inhibitory effects on COX activity. These results pave the way to develop new potent anti-inflammatory compounds to treat numerous pathologies.

## Methods

### Chemicals

**Figure 1:**
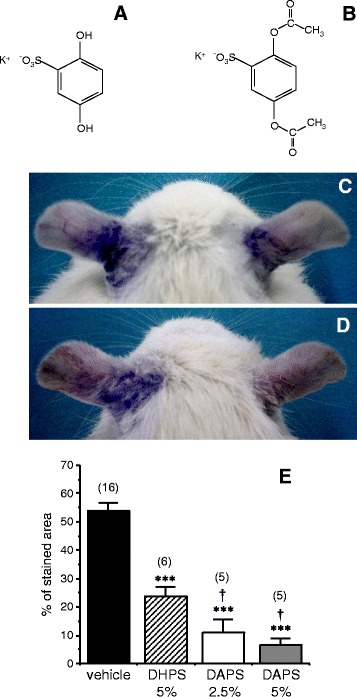
**Chemical structures of potassium 2,5-dihydroxyphenyl sulfonate (DHPS: A) and potassium 2,5-diacetoxyphenyl sulfonate (DAPS: B).** Panels **C** and **D** show representative images illustrating the effects of DHPS **(C)** and DAPS **(D)** on dermatitis of rat ears induced by the application of benzalkonium chloride. After induction of dermatitis in both ears, the right ear was treated topically with DHPS (5% w/v; eq. to 0.22 mmol/ml) or DAPS (5% w/v; eq. to 0.16 mmol/ml), and the left ear with the vehicle alone (glycerol). The extent of dermatitis (vascular hyperpermeability) was revealed by intravenous injection of Evans blue dye. In panel **E** the inhibitory effects of DHPS (5%; eq. to 0.22 mmol/ml) and DAPS (2.5% and 5%; eq. to 0.08 and 0.16 mmol/ml) on dermatitis are quantified, expressing the data as the mean ± SEM of the percentage of blue-stained area relative to the total area of the ear. The number of animals used for each determination is shown in parentheses: ***p < 0.001 *vs.* vehicle, † p < 0.05 vs DHPS by one-factor ANOVA followed by Student-Newmann-Keuls test.

Potassium 2,5-dihydroxyphenyl sulfonate (DHPS) was obtained from Sigma-Aldrich (Saint Louis, MO) and reagent-grade potassium 2,5-diacetoxyphenyl sulfonate (DAPS) was purchased from Aurigene (Bangalore, India). The chemical structures are shown in Figure 1A.

### Animals

Male Sprague-Dawley rats (250 g to 350 g) were obtained from the animal facilities of the Hospital Universitario Ramón y Cajal and they were used for all the animal studies. Studies were performed in accordance with the Declaration of Helsinki, and with the EU guidelines for the handling and care of laboratory animals. All the protocols were approved by the Ethics Committee for Animal Experimentation of the Hospital Universitario Ramón y Cajal (Acta 2/2010). Animals were anesthetized by intraperitoneal injection of 50 mg/kg ketamine and 4 mg/kg diazepam.

### Dermatitis model

Dermatitis was induced by the application of benzalkonium chloride (BZK; 5% w/v solution) in a 1:5 mixture of olive oil:acetone along the back of both ears (40 μl/ear) of anesthetized rats. Thirty minutes after the application of BZK, 40 μl of DHPS (5% w/v) or DAPS (2.5% or 5%) in glycerol were applied topically to the back of the right ear, while glycerol alone (vehicle) was applied to the left ear as a control. Fifteen minutes later, 400 μl of Evans blue dye solution (0.5%; w/v) were injected into the jugular vein, this dye exclusively staining blue the areas of the skin with altered vascular permeability that results in extravasation of the dye. Twenty-four hours after the induction of dermatitis photographs of the extended ears were taken and the stained and total areas of the ear were determined by image analysis (Motic Image Advanced 3.0, Xiamen, China). The stained area of each ear was expressed relative to the total area to determine the percentage of the ear affected by dermatitis.

In another series of experiments, dermatitis was induced on both ears of anesthetized rats as described above and, 30 minutes after applying BZK, 2.5% DAPS or the vehicle alone (glycerol, 40 μl) was applied to both ears. Twenty-four hours after dermatitis induction, the rats were anesthetized and both ears were excised for histological evaluation (left) and to determine myeloperoxidase (MPO) activity (right).

### Histological evaluation

Excised rat ears, gelatin sponges and subcutaneous gliomas were fixed by immersion in 4% paraformaldehyde and embedded in paraffin to obtain 6 μm sections that were deparaffinized in xylene, rehydrated through a series of decreasing ethanol concentrations (100-70%) and finally rinsed in distilled water. The deparaffinized tissue sections were then stained with hematoxylin and eosin for histological examination.

### Myeloperoxidase activity determination

Rat ears were frozen in liquid nitrogen immediately after excision and stored at -80°C until MPO activity was determined as follows. The frozen ears were homogenized in phosphate buffer containing 0.5% hexadecyltrimethylammonium bromide and, after three freeze/thaw/sonication cycles, the samples were centrifuged at 13,000 × g for 20 minutes at 4°C. H_2_O_2_ (0.0005%) and O-dianisidine (0.167 mg/ml) were added to the supernatants, and the absorbance was measured at 460 nm in a spectrophotometer after incubating for 1 hour at room temperature. MPO activity was expressed as the absorbance units per mg of tissue.

### COX inhibition

The inhibitory effects of drugs on cyclooxygenase (COX) activity were determined using a commercially available kit for measuring COX activity (Cayman Chemical, Ann Arbor, MI. Cat # 560131). COX-1 enzyme (ovine) in reaction buffer (0.1 M Tris-HCl [pH 8.0], 5 mM EDTA, 2 mM phenol) containing heme was incubated for 10 min at 37°C in the presence or absence of acetylsalicylic acid (ASA), DHPS or DAPS (1 to 100 μM). Similarly, COX-2 (human recombinant) was incubated for 10 min at 37°C in the same heme-containing reaction buffer in the presence or absence of ASA, DHPS or DAPS (1 to 100 μM). Reactions were triggered with 100 μM arachidonic acid, the reaction substrate, and they were stopped after 2 min by adding HCl. The prostanoid product was quantified by enzyme immunoassay, using PGE_2_ as standard, and measuring the absorbance at 405 nm. The data are expressed as the percentage of COX-1 or COX-2 activity recorded in the absence of inhibitors (100% activity) after subtracting the background activity of heat-inactivated enzyme.

### Determination of coagulation time

Sprague-Dawley rats were anesthetized and a catheter was introduced into the left external jugular vein for saline or drug infusion. The tail was then incised using a disposable surgical blade and bleeding of the tail was monitored by blotting with a filter paper every 30 s. The time from incision to the cessation of bleeding (no blood stains on the paper) was recorded [31]. Bleeding time was measured before and 60 min after intravenous administration of ASA (10 mg/kg), DHPS (10 mg/kg) or DAPS (10 mg/kg).

### Cytokine measurement

Rats were injected with lipopolysaccharide (LPS) from *E. coli* (5 mg/kg, i.p.) or with the vehicle alone (0.9% NaCl, i.p.; controls), and they were subsequently intraperitoneally injected with the vehicle (0.9% NaCl), DHPS (100 mg/kg) or DAPS (100 m/kg). Six hours after LPS injection, blood was collected in dry tubes and the serum isolated by centrifugation. Serum samples were immediately frozen and maintained at -80°C until the serum concentrations of tumor necrosis factor-α (TNF-α), interleukin-6 (IL-6) and interleukin-1ß (IL-1ß) were determined by ELISA (R&D Systems, Abingdon, UK).

### Fibroblast proliferation assays

Inhibition of FGF-mediated mitogenesis was evaluated in murine Balb/C 3 T3 fibroblasts, as described previously [13]. Fibroblasts seeded in 96-well plates were stimulated with FGF-1 (0.64 ng/ml) in the presence of myoinositolhexasulfate (MIHS; 100 μg/ml). Cultures were incubated in the presence or absence (control) of different concentration of DHPS or DAPS (3 μM to 1 mM) and allowed to proliferate for 48 h. Cells were then counted by measuring the total amount of crystal violet fixed by the cell nuclei by determining differential absorbance at 595 nm.

### *In vivo* angiogenesis

Sterile gelatin sponges (1 cm^3^; Curaspon Dental, Clinimed Holding, Zwanenburg, The Netherlands) were moistened with 200 μl of phosphate buffered saline (PBS) solution with or without (controls) 25 μg/ml of heparin and 10 μg/ml of FGF-1, and they were implanted subcutaneously in the dorsal region of the neck of anaesthetized Sprague-Dawley rats as described elsewhere [13]. After implantation, the rats were randomly assigned to groups that received 0.3 ml of vehicle (tap water), DHPS (150 mg/kg) or DAPS (150 mg/kg) twice a day by oral gavage (300 mg/kg/d). In a separate assay, a dose-response relationship of the oral effects of DAPS on *in vivo* angiogenesis was ascertained by administering DAPS at 20, 50, 100 and 300 mg/kg/d. After 7 days, the sponges were removed and sections (6 μm) were obtained and then stained with hematoxylin/eosin as above described. Six random fields (0.176 mm^2^) from each sponge were evaluated to quantify the extent of neovascularization using image analysis software (Motic Images). The effects of the different treatments on vascular density, determined by the number of functional blood vessels (those containing erythrocytes) per field, were evaluated.

### Rat model of subcutaneous glioma

Rat C6 glioma cells were cultured as described previously [32,33]. C6 cells cultured to confluence in 75 cm^2^ flasks were removed and implanted beneath the abdominal skin of anesthetized rats. The presence of tumors in the implantation area was determined 5 days after the implantation of the tumor cells. Rats in which the existence of a tumor was confirmed were randomly assigned for treatment with vehicle (0.9% NaCl), DHPS (100 mg/kg/d) or DAPS (100 mg/kg/d), administered by daily intraperitoneal injection. In an additional series of experiments, oral efficacy of DAPS was evaluated by administration of vehicle (tap water) or DAPS (200 mg/kg/d) by gavage. After 10 days of treatment, either intraperitoneal or oral, the subcutaneous gliomas were removed, photographed, weighed and their volume was calculated according to the formula V = 4/3π•(D/2)•(d/2)^2^ (in mm^3^), where D is the larger diameter and d is the smaller diameter, both expressed in mm.

### Determination of serum lactate dehydrogenase

Blood was collected from anesthetized rats at the moment of subcutaneous glioma extraction and from 8 age-matched rats not bearing tumor (control rats) by cardiac puncture. Blood was drained into dry tubes and the serum isolated by centrifugation. Serum samples were immediately frozen and maintained at -80°C until the assay. Serum lactate dehydrogenase content was determined by using a commercial kit (Architect c Systems, Abbott, Abbott Park, IL, USA) following manufacturer’s instructions.

### Tumoral vascularization and apoptosis

For evaluating tumoral vascularization, tumor sections (6 μm) were obtained and then stained with hematoxylin/eosin as above described. High-resolution images of stained tumor sections were taken and number of blood vessels and areas were determined using morphometric software (Motic). Vascular density was calculated as the total number of functional blood vessels relative to total area of the tumor. Apoptosis was determined by terminal 2′-deoxyuridine-5′-triphosphate (dUTP) nick-end-labeling (TUNEL) assay in deparaffined tissue sections (6 μm) of the subcutaneous gliomas. A fluorescence-based commercial kit was used following manufacturer’s specifications (Promega Biotech Ibérica, Alcobendas, Spain). Percentage of apoptosis was calculated by counting apoptotic cell nuclei (TUNEL-positive cells) relative to total number of cell nuclei in six high-magnification visual fields (x400) for each sample.

## Results

### DAPS is more effective than DHPS in inhibiting dermatitis-induced inflammation

The dermatitis induced in rat ears by 5% BZK was associated with an increase in vascular permeability, evident through the extravasation of Evans blue dye that stains the ears in blue (Figure 1C-D, left ears). Topical treatment with DHPS (5%) or DAPS (5%) markedly inhibited vascular permeability, both treatments diminishing the intensity of the blue staining (Figure 1C-D, right ears). When vascular permeability was quantified as the percentage of area stained, DHPS significantly reduced staining while DAPS induced a more significant reduction, even when administered at half the concentration of DHPS (Figure 1E).

**Figure 2:**
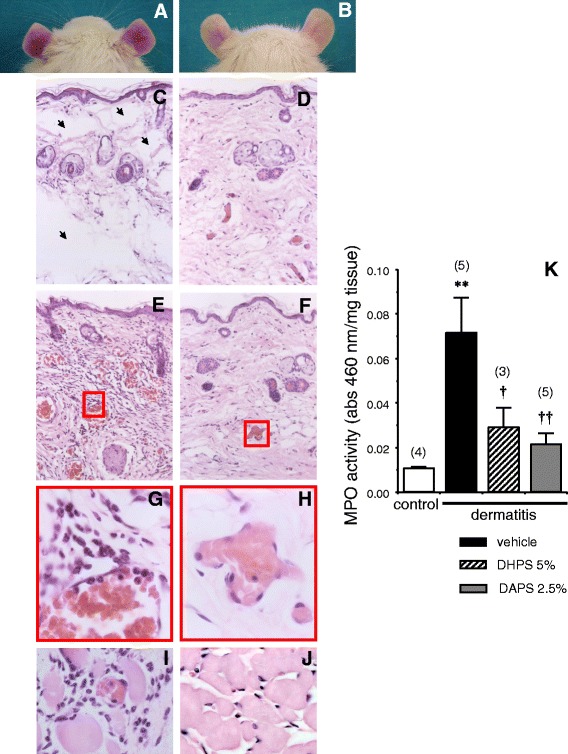
**Representative histology showing the effects of topical treatment with 2,5-diacetoxyphenyl sulfonate (DAPS; 2.5%** **; eq. to 0.08 mmol/ml) on benzalkonium chloride-induced dermatitis in the rat ear.** The first row shows the macroscopic appearance of both ears of a rat treated with vehicle alone (glycerol: **A**) and those of a rat treated with DAPS **(B)**, which evidently reduces the erythema caused by dermatitis. In the second row, the tissue edema on the ear of a rat with dermatitis treated only with glycerol **(C)** was not observed in DAPS-treated rats **(D)**. The third row shows the intense leukocyte infiltration in a glycerol-treated rat ear **(E)**, an effect that was clearly reduced by DAPS treatment **(F)**. Magnification of the boxed area in E and F reveals that in the capillaries of vehicle-treated rats, there are leukocytes adhered to the endothelial cells, which rolled and extravasated to infiltrate the surrounding tissue **(G)**, a feature not observed in the vessels of DAPS-treated rats **(H)**. The infiltration of leukocytes into the erector muscle of the ear in glycerol-treated rats **(I)** was also attenuated by DAPS treatment **(J)**. Magnifications: **C**-**F**, x100; **G**-**H** x400; **I**-**J** x200. Panel **K** shows the effects of topic treatment with 2,5-dihydroxyphenyl sulfonate (DHPS; 5%; eq. to 0.22 mmol/ml), DAPS (2.5%; eq. to 0.08 mmol/ml) or the vehicle alone (glycerol) on the myeloperoxidase (MPO) activity associated with benzalkonium chloride-induced dermatitis on the rat ear. Dermatitis was not induced in the control group. MPO activity is expressed as the mean ± SEM of the absorbance at 460 nm normalized to the weight (mg) of the tissue of the corresponding ear. The numbers of animals used is shown in parentheses: **p < 0.01 *vs.* control; † p < 0.05, †† p < 0.01 *vs.* vehicle by one-factor ANOVA followed by Student-Newmann-Keuls test.

In the absence of dye administration, a marked erythematous response was observed 24 h after the induction of dermatitis with 5% BZK in rat ears (Figure 2A) which was notably prevented by topical application of DAPS (2.5%) (Figure 2B). A detailed histological characterization of the inflammatory response confirmed that topic application of DAPS (2.5%) reduced the extensive skin edema (Figure 2C-D) and leukocyte infiltration (Figure 2E-H) associated with dermatitis in untreated animals. Dermatomyositis was also observed in the erector muscle of the ear after BZK-induced dermatitis (Figure 2I) and likewise, it was prevented by DAPS administration (Figure 2J). The involvement of leukocyte infiltration in this inflammatory response was confirmed by the significant elevation of MPO activity. This increase was prevented by topical administration of DHPS (5%), and more effectively with 2.5% DAPS (Figure 2K).

### DAPS inhibits COX activity and its effects

**Figure 3:**
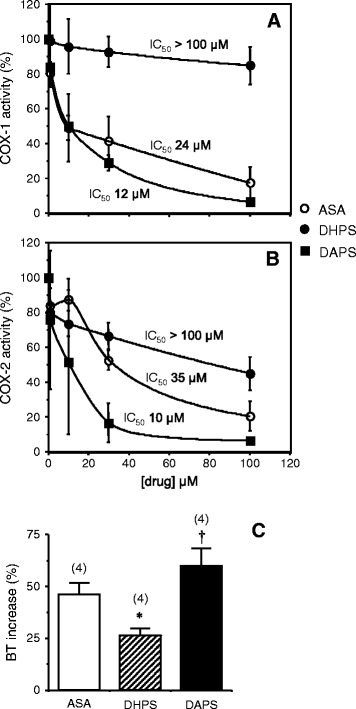
**Effects of acetylsalicylic acid (ASA; 1 to 100 μM), potassium 2,5-dihydroxyphenyl sulfonate (DHPS; 1 to 100 μM) and potassium 2,5-diacetoxyphenyl sulfonate (DAPS; 1 to 100 μM) on cyclooxygenase (COX)-1 (A) and COX-2 (B) activity.** Data are expressed as the percentage of the total COX activity obtained in the absence of inhibitors and the results are the mean ± SEM of two independent experiments performed in duplicate. Panel **C** shows the effects of intravenously administered ASA (10 mg/kg; eq. to 0.05 mmol/kg), DHPS (10 mg/kg; eq. to 0.04 mmol/kg) and DAPS (10 mg/kg; eq. to 0.03 mmol/kg) on bleeding time (BT) in anesthetized rats. The data are expressed as the mean ± SEM of the percentage of increase in BT with respect to the basal determination for each animal. The numbers of rats used for the measurements are indicated in parentheses: *p < 0.05 *vs.* ASA; † p < 0.05 *vs.* DHPS, by one-factor ANOVA followed by Student-Newmann-Keuls test.

DHPS (1 to 100 μM) had no significant inhibitory effect on COX activity, whereas the acetyloxy derivative DAPS (1 to 100 μM) induced significant concentration-dependent inhibition of both COX-1 and COX-2 (Figure 3A-B). In fact, DAPS was as potent as ASA in inhibiting COX-1 activity, and even more efficient than ASA in inhibiting COX-2 (Figure 2A-B).

An increase in bleeding can be used to estimate the decrease of thromboxane production by platelets as a consequence of COX inhibition in these organelles. The anti-thrombotic capacity of DAPS (10 mg/kg, i.v.) was equivalent to, or greater than, that induced by intravenous administration of ASA (10 mg/kg), and it was twice that observed for the parent molecule, DHPS (10 mg/kg, i.v.: Figure 3C).

### DAPS reduces pro-inflammatory cytokine levels

**Figure 4:**
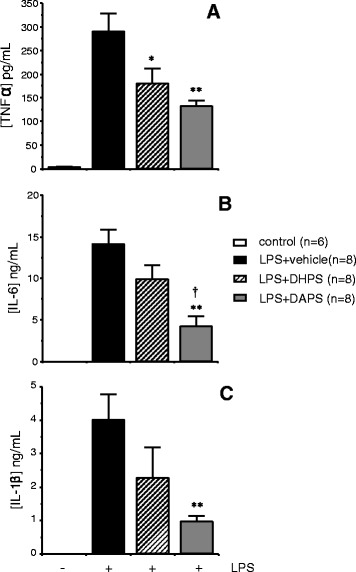
**Effects of intraperitoneal injection of potassium 2,5-dihydroxyphenyl sulfonate (DHPS; 100 mg/kg; eq. to 0.44 mmol/kg) or potassium 2,5-diacetoxyphenyl sulfonate (DAPS; 100 mg/kg; eq. to 0.32 mmol/kg) on the lipopolysaccharide (LPS)-induced increase in tumor necrosis factor-α (TNF-α; A), interleukin-6 (IL-6; B) and interleukin-1ß (IL-1ß; C) concentrations in rat serum.** Serum samples were obtained 6 h after LPS injection (5 mg/kg; i.p.) and the data are expressed as the mean ± SEM concentration of cytokine in pg/ml or ng/ml: * p < 0.05, **p < 0.01 *vs.* LPS + vehicle, † p < 0.05 vs. LPS+DHPS, by one-factor ANOVA followed by Student-Newmann-Keuls test.

Since, in addition to mediating local inflammatory processes, FGF and COX could have an impact on systemic inflammation, we evaluated the effect of DHPS (100 mg/kg; i.p.) and DAPS (100 mg/kg; i.p.) on the level of TNF-α, IL-6 and IL-1ß in a rat model of systemic inflammation. Precisely, the marked increase of the serum levels of TNF-α caused by intraperitoneal injection of LPS (5 mg/kg) was significantly attenuated by DHPS, and to a larger extent, by DAPS administration (Figure 4A). LPS also markedly increased serum concentrations of IL-6 and IL-1ß. Although DHPS showed a trend to reduce the elevation of these cytokines, only DAPS was able to significantly reduce the increase in IL-6 and IL-1ß caused by LPS (Figure 4B-C).

### Acetylated DHPS inhibits the mitogenic activity of FGF *in vitro* and displays enhanced anti-angiogenic activity *in vivo*

**Figure 5:**
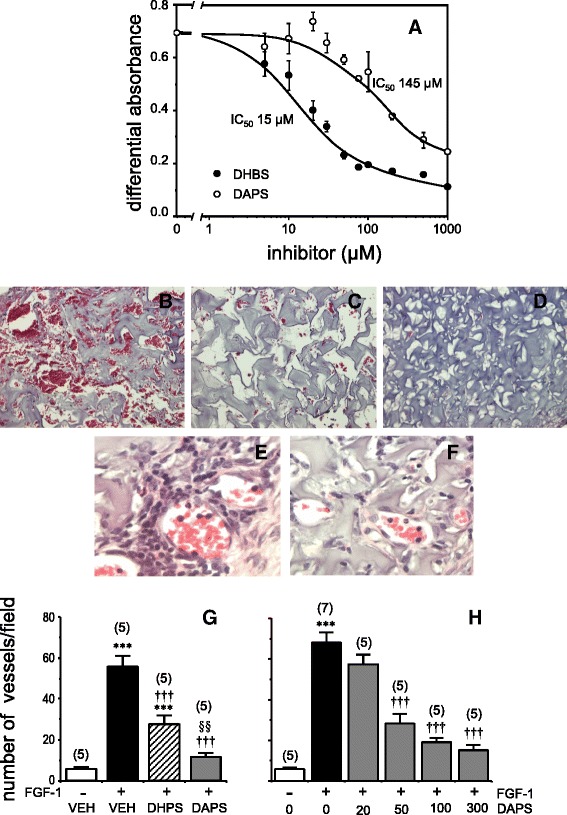
**Inhibition of FGF-1-induced mitogenesis in vitro and angiogenesis** ***in vivo*** **by potassium 2,5-dihydroxyphenyl sulfonate (DHPS) and potassium 2,5-diacetoxyphenyl sulfonate (DAPS).** Panel **A** shows the inhibition of mitogenesis induced by fibroblast growth factor (FGF)-1 in quiescent Balb/c 3T3 fibroblasts treated with DHPS or DAPS. Representative microphotographs show how oral administration of the vehicle alone (VEH; tap water: **B**), DHPS (300 mg/kg/day; eq. to 1.32 mmol/kg/d; **C**) and DAPS (300 mg/kg/day; eq. to 0.96 mmol/kg/d; **D**) affect FGF-1-induced angiogenesis in gelatin sponges subcutaneously implanted in rats for 7 days. In the same assay, intense leukocyte extravasation and infiltration can be observed in sponges containing FGF-1 when they are removed from vehicle-treated rats **(E)** but not from DAPS-treated rats **(F)**. The extent of neovascularization detected in phosphate buffered saline (PBS)- and FGF-1-containing sponges removed from rats treated with vehicle, DHPS or DAPS can be quantified **(G)**. In a separate assay **(H)** the dose-response relationship of the effects of orally administered DAPS is shown (20 to 300 mg/kg/day; eq. to 0.06 to 0.96 mmol/kg/d), expressing the data as the mean ± SEM number of functional vessels per field, determined in 6 randomly acquired fields per specimen. The number of rats used for each measurement is indicated in parentheses: ***p < 0.001 *vs.* neovascularization in the absence of FGF-1; ††† p < 0.001 *vs.* FGF-1 + VEH; §§ p < 0.01 *vs.* FGF-1 + DHPS by one-factor ANOVA followed by Student-Newmann-Keuls test. Magnifications: **B**-**D**, x200; **E**-**F**, x400.

DAPS inhibited FGF-induced proliferation of 3T3 fibroblasts *in vitro* (Figure 5A), although with a higher IC_50_ than DHPS. This result is not completely surprising, given the tight fit of the latter compound into a narrow pocket at the surface of FGF-1 [13]. However, DAPS is still a better FGF inhibitor than other compounds used as leads in the DHPS discovery process, which have been widely used to inhibit FGF-induced angiogenesis [34].

FGF-1 induced extensive angiogenesis in subcutaneously-implanted gelatin sponges, which was drastically attenuated by oral administration of DAPS or DHPS (Figure 5B-D), although more so by DAPS (Figure 5G). The oral efficacy of DAPS to inhibit FGF-induced angiogenesis in this model and the dose-dependency of this effect is shown in Figure 5H. This FGF-induced neovascularization is associated with leukocyte infiltration (Figure 5E). The strong inhibition of FGF-induced angiogenesis by DAPS was accompanied by a clear reduction of FGF-induced leukocyte infiltration (Figure 5F).

### Acetylated DHPS displays superior anti-tumoral activity *in vivo*

**Figure 6:**
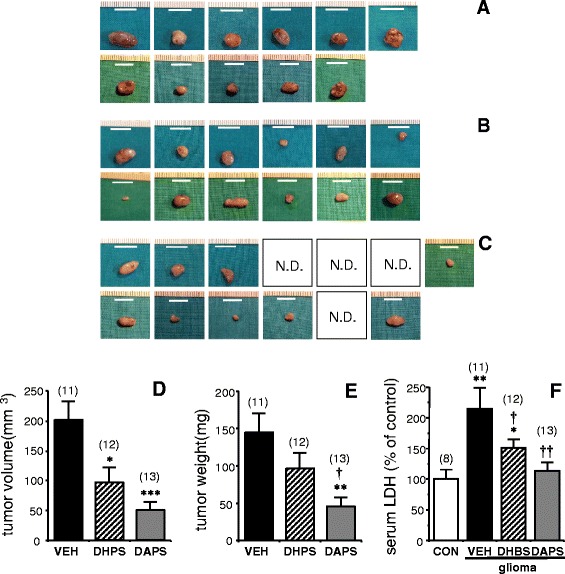
**Effects of intraperitoneal administration of potassium 2,5-dihydroxyphenyl sulfonate (DHPS) and potassium 2,5-diacetoxyphenyl sulfonate (DAPS) on the progression of tumors established in rats by subcutaneous implantation of rat glioma C6 cells (5 × 10** ^**5**^ **cells).** Photographs show tumors removed from rats treated with vehicle (0.9% NaCl; n = 11; **A**), DHPS (100 mg/kg/d; eq. to 0.44 mmol/kg/d, i.p. for 10 days; n = 12; **B**), or DAPS (100 mg/kg/d, eq. to 0.32 mmol/kg/d, i.p. for 10 days; n = 13; **C**). Treatment began once the presence of a tumor was verified on the fifth day after glioma cell implantation. Tumors were removed after 10 days of treatment and the empty spaces in panel **C** correspond to tumors that had completely regressed after DAPS treatment (N.D., not detected). A 10 mm bar is displayed in each image. Panels **D** and **E** show the quantification and comparison of the volumes and weights, respectively, of the tumors developed in rats treated with vehicle (VEH), DHPS or DAPS. The data are expressed as the mean ± SEM. numbers of animals are shown in parenthesis. *p < 0.05; **p < 0.01; ***p < 0.001 *vs.* VEH; † p < 0.05 *vs.* DHPS, by one-factor ANOVA followed by Student-Newman-Keuls test. Panel **F** shows the effects of subcutaneous glioma development and the treatment with DHBS and DAPS on serum concentrations of lactate dehydrogenase (LDH) in rats. The data are expressed as the mean ± SEM of the percentage of serum LDH detected in control, not bearing glioma, rats (CON). Numbers of animals are shown in parenthesis. *p < 0.05; **p < 0.01; *vs.* CON; † p < 0.05 *vs.* VEH, by one-factor ANOVA followed by Student-Newman-Keuls test.

Considering the above confirmed capacity of DHPS and DAPS to inhibit angiogenesis and inflammation and given the key roles of these processes in carcinogenesis, the effects of DHPS and DAPS on tumoral progression were evaluated. To estimate the relative anti-tumoral potential of these two compounds, we used a well-known heterotopic model of glioma. As seen in Figure 6A-C, gliomas in rats treated for 10 days with vehicle (0.9% NaCl) were larger than those in rats treated with DHPS (100 mg/kg/day; i.p.) or DAPS (100 mg/kg/day; i.p.). Rats that received DHPS also exhibited larger tumors than those treated with DAPS, and indeed, four rats that received DAPS were tumor-free by the end of treatment, despite the fact that all the rats developed comparable tumors prior to treatment. By contrast, none of the tumors were fully eliminated in the animals administered the vehicle alone or DHPS. Quantification of the tumor volume confirmed that tumor progression was significantly inhibited in DHPS-treated rats, an effect that was further enhanced in DAPS-treated rats (Figure 6D). Evaluation of the tumor weight yielded similar results, although the reduction in weight was only significant in rats treated with DAPS (Figure 6E). This attenuated effect may be explained by the existence of necrotic processes in vehicle-treated tumors, a frequently-observed phenomenon that diminishes tumor density as the tumor volume increases. In fact, the serum concentrations of lactate dehydrogenase (LDH), which is a marker of tumoral necrosis [35,36], are significantly increased in vehicle treated rats with subcutaneous gliomas when compared to healthy not bearing glioma rats. Serum LDH levels in glioma implanted rats were significantly reduced by DHPS treatment and normalized by treating with DAPS (Figure 6F).

**Figure 7:**
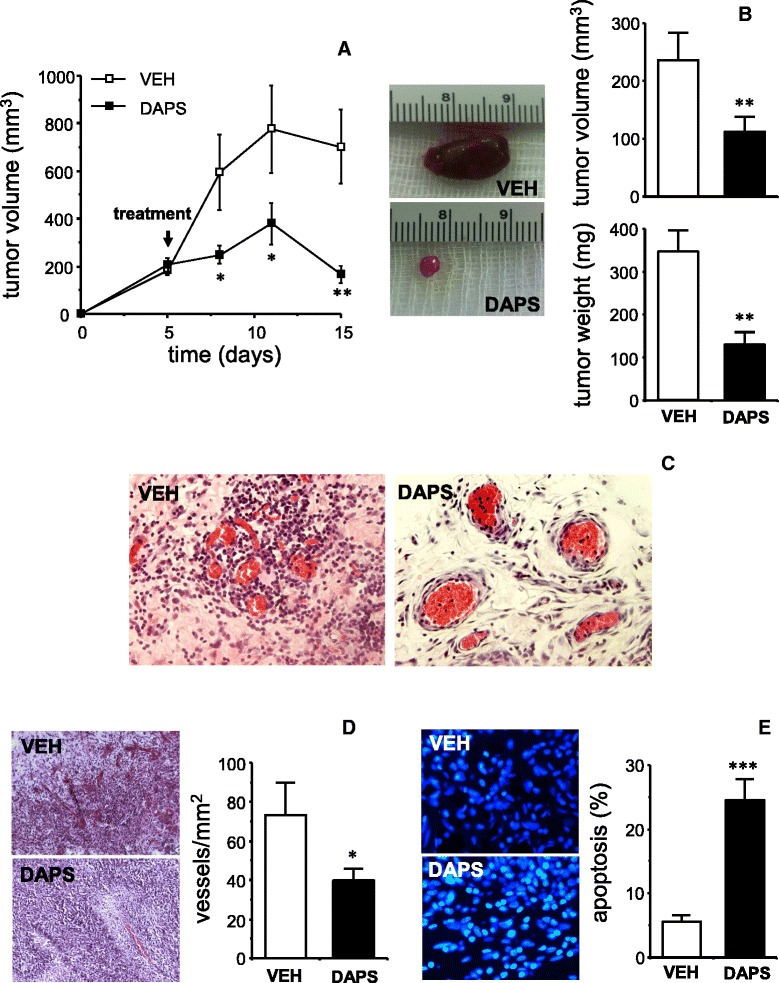
**Effects of oral administration of potassium 2,5-diacetoxyphenyl sulfonate (DAPS) on the progression, angiogenesis and apoptosis of tumors established in rats by subcutaneous implantation of rat glioma C6 cells (5 × 10** ^**5**^ **cells).** Treatment began once the presence of a tumor was verified on the fifth day after glioma cell implantation (arrow in panel **A**). Tumors were removed after 10 days of treatment with vehicle (VEH, tap water; n = 8) or DAPS (200 mg/kg/d; eq. to 0.73 mmol/kg/d; n = 10) by oral gavage. Panel **A** shows the time-course of subcutaneous glioma size in rats treated with vehicle or DAPS. Panel **B** shows representative images of macroscopic aspect of tumors excised from rats treated with vehicle or DAPS while the quantification and comparison of the volumes and weights are shown in the same row. Panel **C** shows representative images illustrating the intense leukocyte extravasation and infiltration observed in peritumoral vessels of gliomas obtained from vehicle-treated rats that is markedly reduced in gliomas from rats treated with DAPS. Panel **D** shows representative images illustrating the intense vascularization in tumors from vehicle-treated rats which is notably reduced in DAPS-treated rats, as confirmed by functional vessel quantification displayed at the right side. Panel **E** shows representative images illustrating the scarce presence of apoptotic nuclei detected by TUNEL assay in sections of tumors from vehicle-treated rats that markedly increased in DAPS-treated rats, as confirmed by quantification of the percentage of tumoral apoptotic nuclei displayed at the right side. The data are expressed as the mean ± SEM: *p < 0.05; **p < 0.01; ***p < 0.001 *vs.* VEH by unpaired Student’s *t* test.

Furthermore, DAPS displayed anti-tumoral efficacy also when orally administered (Figure 7). Oral administration of DAPS (200 mg/kg/d) resulted in reduced progression of subcutaneous gliomas induced by C6 cell implantation in rats (Figure 7A). This was confirmed by the significant reduction in tumor volume and weight after the treatment with DAPS (Figure 7B). A strong leukocyte infiltration from peritumoral vessels was observed in vehicle-treated rats that was notably reduced in rats treated with DAPS (Figure 7C). Inhibition of tumoral progression by oral DAPS was accompanied by a reduction of tumor vascularization (Figure 7D) and an increase in tumor apoptosis (Figure 7E). Oral treatment with DAPS for 10 days did not cause signs of toxicity since weight gain of animals treated with DAPS was not different from that observed in vehicle treated animals. Baseline weights just before C6 implantation were 332 ± 12 g and 319 ± 14 g for vehicle- and DAPS-treated groups, respectively, while final weights 15 days afterwards, including the 10-days treatment period, were 374 ± 15 g and 373 ± 13 g for vehicle- and DAPS-treated groups, respectively.

## Discussion

In the present study we demonstrate that acetate esterification of the two hydroxyl groups of DHPS generates a derivative, DAPS, which inhibits FGF and COX, and that exhibits anti-coagulant, anti-inflammatory (local and systemic), anti-angiogenic and anti-tumoral activities that are considerably more potent than those of DHPS. These enhanced properties of DAPS were not totally unexpected, as they are regularly observed after COX inhibition, and the modifications introduced confer DAPS with the typical chemical features of phenolic COX inhibitors [29,30]. However, the fact that some of these properties are already present in DHPS, which does not inhibit COX, is indeed surprising. Nonetheless, gentisic acid was recently described as the head of a family of potent FGF inhibitors, the most active member of which is DHPS [13]. Accordingly, these unexpected properties may merely be a consequence of FGF inhibition.

The role of FGFs in inflammatory processes has received little attention due to their important mitogenic activities in many biological processes, and their modulatory role during embryonic development and differentiation. Nonetheless, there is significant evidence that FGFs are involved in inflammation. As early as 1990, a T-cell-dependent increase in FGF-1 expression was described in the inflamed joints of patients with rheumatoid arthritis, and it was directly correlated with the extent and intensity of inflammation [37]. Subsequently, upregulation of FGF-1 was described in allografts undergoing chronic rejection [38] and in renal inflammation [39], while elevated levels of circulating FGF-2 were reported in patients with inflammatory bowel disease [40] and eosinophilic esophagitis [41]. The pericardial fluid of patients with inflammatory pericardial disease also contains increased levels of FGF-2 [42]. The anti-inflammatory activity of DHPS in dermatitis model confirms the contribution of FGF to inflammation. The added ability of the acetyloxy derivative DAPS to inhibit COX activity together with its inhibitory activity on FGF would explain the enhanced anti-inflammatory properties of this compound over its parental molecule. In addition to its key role in local inflammatory responses, COX activity could contribute to systemic inflammation. In fact, inhibition of COX results in decreased production of the pro-inflammatory cytokines, TNF-α, IL-6 and IL-1ß in LPS-stimulated macrophages [43] and aspirin administration reduces plasma IL-1ß and IL-6 in patients with metabolic syndrome [44]. Thus, the potent inhibitory effect of DAPS on COX activity likely accounts for the marked reduction of inflammatory cytokine elevation induced by LPS in rats. However, the non-significant reduction of IL-6 and IL-1ß and the significant reduction of TNF-α increase in LPS-stimulated rats by DHPS suggest a positive modulation by FGF in inflammatory cytokine elevation. This would be consistent with evidences showing that FGF-1 stimulates cytokine production in Jurkat T-cells via NF-кB activation [45], while FGFR2 activation by FGF-1 potentiates the secretion of TNF-α and IL-6 in LPS/INFγ-stimulated human astrocytes and microglia [46].

It may be somewhat surprising that an FGF inhibitor can exhibit so good a safety profile as DHPS (dobesilate) [10], given the broad spectrum of physiological activities in which FGFs are involved [47]. FGFs have been described in most adult tissues derived from the embryonic mesoderm and neuroectoderm, where they are tightly bound to the sulfated glycosaminoglycans of the extracellular matrix, sometimes at very high levels [48]. Although initially described as FGF traps and protectors, these proteoglycans were later found to participate in FGF signaling [49,50]. Thus, under normal physiological conditions FGFs do not act as signaling molecules in solution but rather, as solid-phase-like growth factors [51]. As long as FGFs remain part of the extracellular matrix, they cannot be inhibited by dobesilate, which has an affinity constant for FGF approximately 3,000 times lower than that of the sulfated glycosaminoglycans of the extracellular matrix, which can reach extraordinarily high local concentrations [13]. However, the physiological FGF signaling system can be subverted when high concentrations of free FGF accumulate, resulting in serious physiological disturbances, either through uncontrolled synthesis or mobilization by heparanases and other specialist proteins [52-54]. By contrast, free FGF is efficiently inhibited by dobesilate [13], which mainly provokes the inhibition of pathological FGF, leaving the physiological FGF pool relatively unchanged.

Our *in vivo* assays demonstrated that despite being a poorer *in vitro* inhibitor of FGF than DHPS, DAPS is more effective in inhibiting FGF-1-induced angiogenesis. However, it should be noted that angiogenesis in the implanted sponges appeared to be a consequence of FGF-evoked inflammation, and that inflammatory chemokines and inflammatory cells are early mediators of FGF-driven angiogenesis both *in vivo* and during neovascularization, according to the expression profile of FGF-stimulated microvascular cells [14,55,56]. Indeed, the induction and inhibition of FGF-driven angiogenesis *in vivo* was consistently associated with an increase and decrease in inflammatory cell infiltration of the implanted gelatin sponges, respectively [29]. The superiority of DAPS as an anti-angiogenic drug is probably due to its COX-related anti-inflammatory activity, a view consistent with the active role of COX and PG synthesis in angiogenesis [57,58].

The efficacy of DHPS in inhibiting glioma growth in both heterotopic and orthotopic models has been demonstrated previously [13,18]. Unlike xenograft models in immunosuppressed animals, rats bearing gliomas in these models have an intact immune system, which is an important distinction as inflammation is generally accepted to play a critical role in tumorigenesis [59,60]. Most solid malignancies trigger intrinsic inflammatory responses that promote a pro-tumorigenic microenvironment [61]. Furthermore, COX and the products of its activity are specifically involved in carcinogenesis [62-64]. In fact, COX inhibition has been recently reported to potentiate anti-angiogenic cancer therapy in preclinical models [65]. COX was indeed proposed to be a therapeutic target for the treatment of gliomas [66,67]. The participation of COX in tumoral processes may explain the superior efficacy of DAPS in inhibiting the progression of subcutaneous gliomas, which in some cases even resulted in the complete regression of established tumors. In addition, it should be noted that the efficacy of DAPS to inhibit angiogenesis and tumor progression is also demonstrated when orally administered, increasing its potential therapeutic relevance. Furthermore, oral treatment with DAPS does not prevent normal weight gain in rats which points to the absence of dramatic toxicity under these experimental conditions. Histological analyses of the tumors suggest that the inhibition of tumoral progression by DAPS is related to its capacity to reduce angiogenesis and to promote apoptosis, two hallmarks of glioma progression [24]. FGF contributes to both processes as demonstrated by the inhibition of angiogenesis and apoptosis by DHPS in orthotopic experimental gliomas [18]. In fact, an intense leukocyte infiltration is observed in peritumoral vessels of subcutaneous gliomas However COX activity seems to be involved in angiogenesis and glioma cell survival (escape from apoptosis) [68,69] supporting dual FGF/COX inhibition by DAPS as a reasonable strategy to inhibit glioma progression.

## Conclusions

The present results support a role for FGF in inflammation and indicate a potential therapeutic role of FGF inhibitors such as DHPS in inflammatory processes. In addition, we demonstrate that a simple modification of DHPS by acetylating hydroxyl residues in the phenyl ring yields a new compound, DAPS, which inhibits COX while maintaining substantial FGF inhibitory activity, a characteristic that enhances its anti-inflammatory activity. This modification converts DAPS into a superior oral inhibitor of angiogenesis and tumor progression than DHPS and represents a reasonable strategy to combat tumoral processes as well as other inflammation- and angiogenesis-related diseases.
